# Supplementary material for: Characterization of Microbotryum lychnidis-dioicae Secreted Effector Proteins, Their Potential Host Targets, and Localization in a Heterologous Host Plant
Source: J Fungi (Basel). 2024 Mar 30;10(4):262. doi: 10.3390/jof10040262 (PMC11051474; doi:10.3390/jof10040262)
Supplement: Supplementary file 1 [file jof-10-00262-s001.zip › jof-2898344-supplementary.pdf]

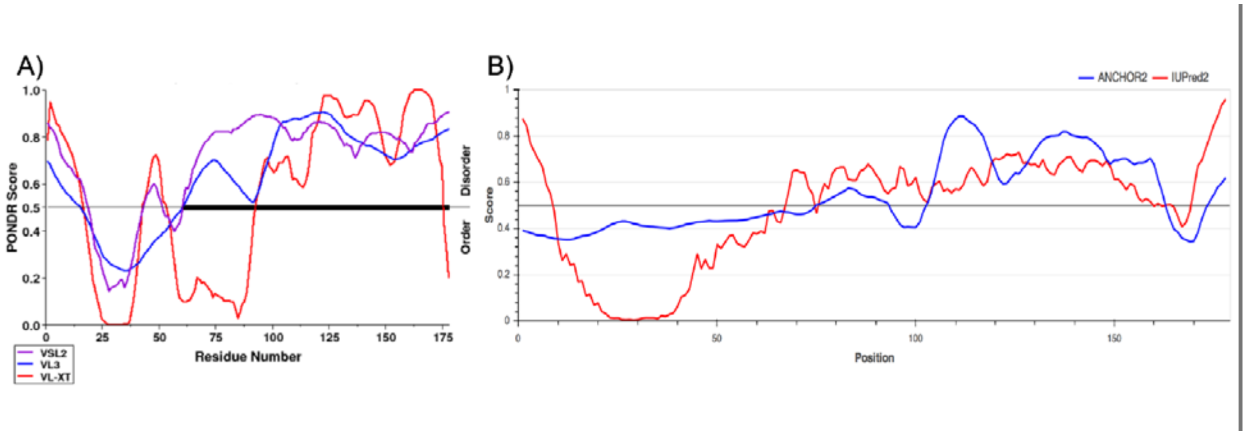

Figure S1. Prediction of disorder in the protein sequence for MVLG_02245 using A) PONDR and B) IUPred2A.

a) MVLG_02245

*MTSQVRMQVESRAQRRAGAYASMRLLLALVFALCTLAHLPTTSA*APLASEQISSGLVF
RQEPPRWLQFSRPHEKVSHQGKDHLDWKNTSPSPFTSSEPSRRVKRDEMWEQYIEG
DEIDGEKSEDVRAGDPDVAGDEVLTDTEIAGGADEAGEGSTGEKWWQARRRLRERR
SATTRVVP

b) MVLG_06175

*MWTSSIVQAALLFAVIVLYSSPVVAWA*FCPFGKTAEHMAICSSLCRMRCYDPNSGTSNSTCRNA
CTGQYHVSRSLNAADQCMQQCDRFTKDKKKQGEGKLEHKRCLHKCTDWFFPLNL

c) MVLG_05122

*MLFKVSAALVLAGLSLGASA*LPSMSTESRAQPSPSSNKSPYGRTGYIDSPADRKTTTYKVGDKI
HFVYTSAPATYFVDVSLMLANGSQSFQLANRLTGSSMISNDANARAYFRMPENLKTIATELLAAS
QDEHSGAMKNNNCILAYLIAKETQNGQYGLVGNLETKQAIAISM

Figure S2. Amino acid sequences for a) MVLG_02245; b) MVLG_06175; and c) MVLG_05122. The signal peptide, as predicted by SignalP is indicated in italics and red for each protein.

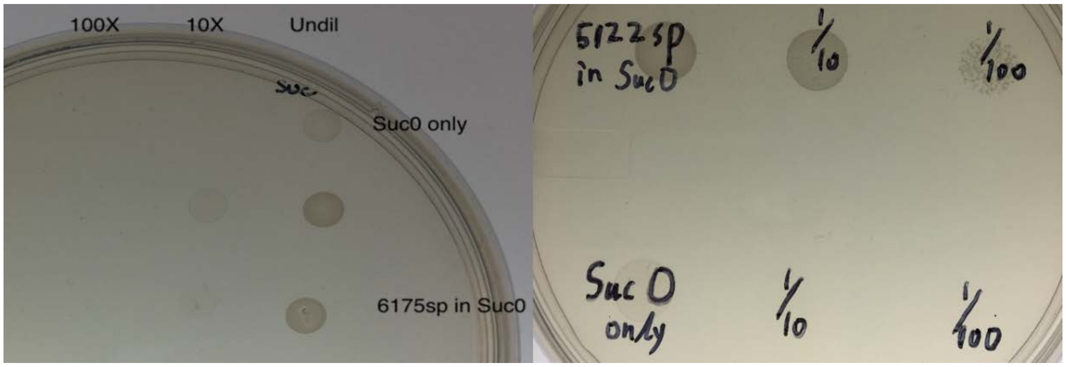

Figure S3. Yeast secretion trap assay. *S. cerevisiae* SEY 6210 cells transformed with pYSTO-0 encoding either the secretion signal sequence of *MVLG_06175* (Left) or *MVLG_05122* (Right) upstream of *SUC2* were able to proliferate on agar where leucine was absent and sucrose was the only carbon source, indicating both MVLG_06175 and MVLG_05122 are normally secreted from fungal cells. Suc0 only, the pYSTO-0 vector without the signal sequence and start codon, as a negative control; 6175SP/5122SP in Suc0, the vector with the signal sequence of *MVLG_06175*/*MVLG_05122*; Undil, undiluted; 10X and 1/10, 10-fold dilution; 100X and 1/100, 100-fold dilution

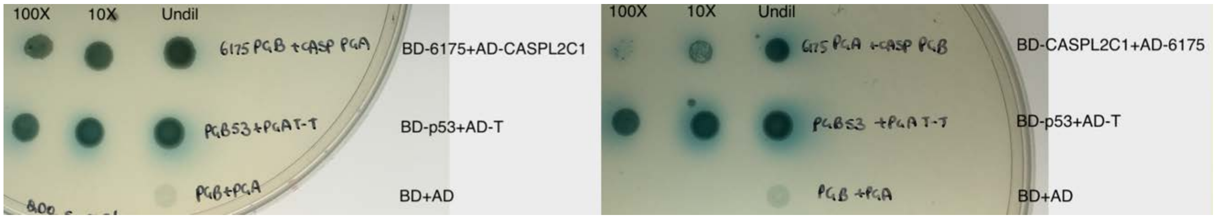

(a) (b)

Figure S4. Y2H spot test on *MVLG_06175*. In order to reconfirm the protein-protein interactions between the fungal and host plant proteins, Y2H spot tests with and without vector-switch were conducted for comparison. (a) The fungal gene *MVLG_06175* was first expressed from the bait vector bearing a binding domain (BD) and plant gene *CASPL2C1* was expressed from the prey vector bearing an activating domain (AD). The bait vector and prey vector were transformed into AH109 and Y187 yeast strains, respectively. The two yeast strains were mated and spotted onto agar containing QDO medium/X-α-gal + 3AT (50 mM). (b) To ensure that interactions were not due to artifacts of the particular vectors, both genes were amplified and inserted into the opposite vector, followed by transformation and mating of yeast. The result showed MVLG_06175 and CASPL2C1 interacted although they were expressed from the opposite vector, suggesting the protein-protein interactions are genuine. BD-p53+AD-T, the positive control for Y2H interactions; BD+AD, bait and prey vectors with no inserts, as a negative control; Undil, undiluted; 10X and 100x, 10-fold and 100-fold of dilution.

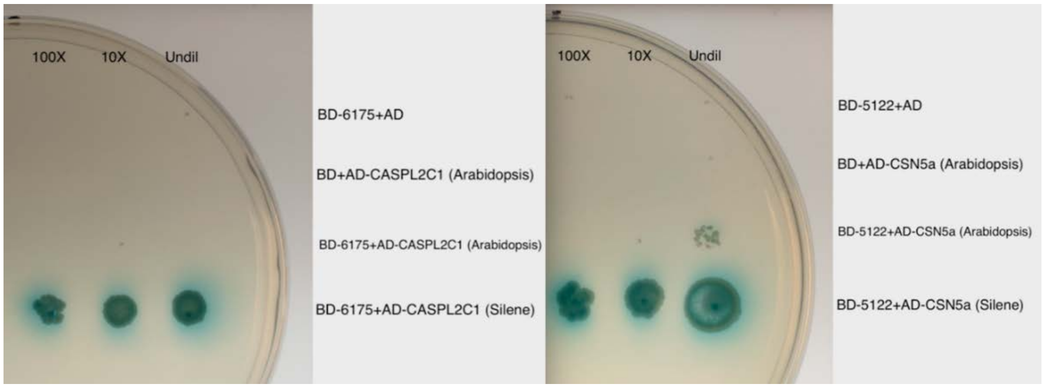

(a) (b)

Figure S5. Y2H spot test on protein-protein interactions between plant proteins of *A. thaliana* and fungal proteins MVLG_06175 and MVLG_05122. To confirm the protein-protein interactions in the model plant *A. thaliana*, *CASPL2C1* and *CSN5a* of the plant were inserted into the prey vector bearing the transcriptional activation domain (AD). The modified prey vectors were transformed into the Y187 yeast strain. After the transformation, the Y187 yeast strain was mated with the AH109 yeast strain bearing the bait vector encoding either *MVLG_06175* or *MVLG_05122* in QDO medium/X-α-gal + 3AT (25 mM). (a) The protein-protein interaction between MVLG_06175 and CASPL2C1 of *A. thaliana* was absent, but (b) the interaction was observed between MVLG_05122 and CSN5a of the plant. BD-6175+AD-CASPL2C1 (Silene) and BD-5122+AD-CSN5a (Silene), positive controls; BD-6175+AD, BD+AD-CASPL2C1 (Arabidopsis), BD-5122+AD, and BD+AD-CSN5a (Arabidopsis), one of the two mating yeast strains carries bait or prey vectors with no insertions, as negative controls; Undil, undiluted; 10X and 100X, 10 folds and 100 folds of dilutions.

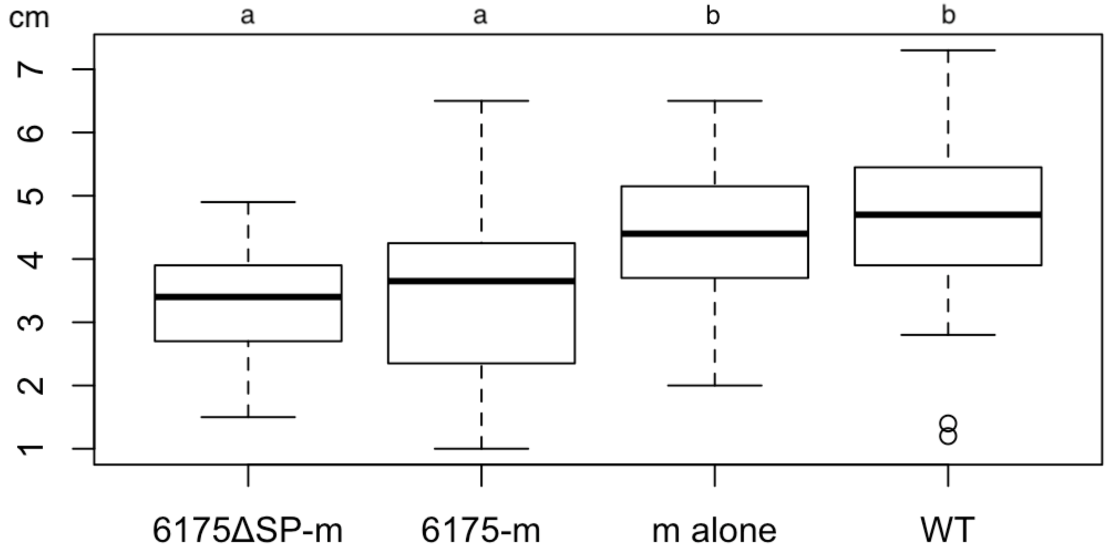

Figure S6: Box-and-Whisker plot showing rosette diameter among transgenic *A. thaliana* lines. The experimental groups are plants transformed with the *MVLG_06175* (with and without the signal sequence) linking to the *mCherry* fluorescence gene. The control groups are plants transformed with *mCherry* fluorescence gene alone and wild type *Col-0* (WT). The t-test analysis demonstrates plant lines expressing MVLG_06175, both with and without the signal sequence, are significantly smaller in rosette diameter than the controls. 6175ΔSP-m, MVLG_06175ΔSP-mCherry. 6175-m, MVLG_06175-mCherry. m alone, mCherry alone. 6175ΔSP-m vs m alone, $p < 0.0001$****. 6175-m vs m alone, $p < 0.001$***. 6175ΔSP-m vs WT, $p < 0.0001$****. 6175-m vs WT, $p < 0.01$**

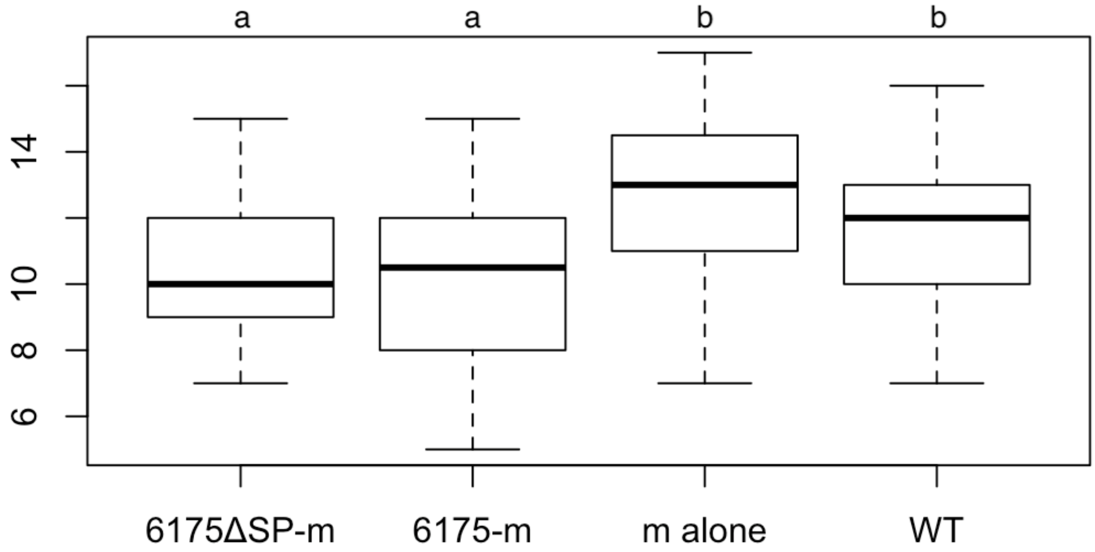

Figure S7: Box-and-Whisker plot showing leaf quantity among transgenic *A. thaliana* lines. The experimental groups are plants transformed with the *MVLG_06175* (with and without the signal sequence) linking to the *mCherry* fluorescence gene. The control groups are plants transformed with *mCherry* fluorescence gene alone and wild type *Col*-0 (WT). The t-test analysis demonstrates plant lines expressing MVLG_06175, both with and without the signal sequence, are significantly smaller in leave quantity than the controls. 6175ΔSP-m, MVLG_06175ΔSP-mCherry. 6175-m, MVLG_06175-mCherry. m alone, mCherry alone. 6175ΔSP-m vs m alone, $p < 0.001$***. 6175-m vs m alone, $p < 0.001$***. 6175ΔSP-m vs WT, $p < 0.05$*. 6175-m vs WT, $p < 0.05$*
